# Near-Infrared Light-Emitting Diodes Based on RoHS-Compliant InAs/ZnSe Colloidal Quantum Dots

**DOI:** 10.1021/acsenergylett.2c02070

**Published:** 2022-10-06

**Authors:** Manuela De Franco, Dongxu Zhu, Aswin Asaithambi, Mirko Prato, Eleftheria Charalampous, Sotirios Christodoulou, Ilka Kriegel, Luca De Trizio, Liberato Manna, Houman Bahmani Jalali, Francesco Di Stasio

**Affiliations:** ∇ Dipartimento di Chimica e Chimica Industriale, Università degli Studi di Genova, Via Dodecaneso 31, 16146 Genova, Italy; ^‡^Photonic Nanomaterials, ^§^Nanochemistry, ^∥^Functional Nanosystems, and ^⊥^Materials Characterization Facility, Istituto Italiano di Tecnologia, Via Morego 30, 16163 Genova, Italy; ^#^Inorganic Nanocrystals Laboratory, Department of Chemistry, and ^⊗^Experimental Condensed Matter Physics Laboratory, Department of Physics, University of Cyprus, 1678 Nicosia, Cyprus

## Abstract

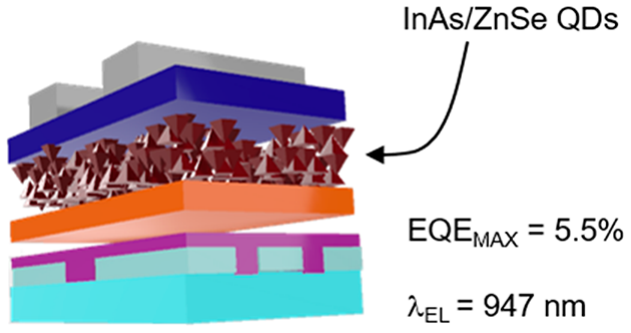

We demonstrate efficient, stable, and fully RoHS-compliant near-infrared (NIR) light-emitting diodes (LEDs) based on InAs/ZnSe quantum dots (QDs) synthesized by employing a commercially available amino-As precursor. They have a record external quantum efficiency of 5.5% at 947 nm and an operational lifetime of ∼32 h before reaching 50% of their initial luminance. Our findings offer a new solution for developing RoHS-compliant light-emitting technologies based on Pb-free colloidal QDs.

Light-emitting diodes (LEDs) operating in the near-infrared (NIR) range (700–1700 nm) are of importance for telecommunications and optical diagnostic as well as for remote sensing and *in vivo* imaging.^1^ So far, all reported efficient NIR LEDs are based on PbS quantum dots (QDs)^2,3^ or on a Pb-containing halide perovskite host matrix.^4^ However, due to the European Union’s “Restriction of Hazardous Substances” (RoHS) directive, these toxic materials cannot be approved for optoelectronic applications.^1^ For this reason, the search for appropriate Pb-free and RoHS-compliant compositions is an important focus in the development of QD-based NIR LEDs.

Colloidal indium arsenide (InAs) QDs are among the few RoHS-compliant materials having high potential for application in NIR optoelectronic devices. Yet, the integration of InAs QDs into optoelectronic devices such as NIR LEDs lags far behind the PbS-based ones. The main reasons for such limited advancement are the complex synthesis and poor optical properties of InAs QDs,^5^ along with limited material design and device engineering when using such material.^6,7^ Electroluminescence (EL) from InAs QDs films has been demonstrated only very recently in QDs coated with multiple shells, a relatively elaborate architecture based on a In(Zn)As/In(Zn)P/GaP/ZnS system synthesized via pyrophoric and expensive tris(trimethylsilyl)phosphine and tris(trimethylsilyl)arsine precursors.^8^ The NIR-emitting LEDs fabricated using such QDs had an external quantum efficiency (EQE) of 4.6% at 850 nm. It is hence evident that many fundamental challenges (synthesis, operating wavelength, EQE, and operation stability) need to be addressed before InAs QD-based NIR LEDs can gain equivalent attention in NIR technology as the “state of the art” PbS-based devices.

In this work, we demonstrate a fully RoHS-compliant QD NIR LED operating at 947 nm based on InAs/ZnSe core/shell QDs. Key ingredients in our device are InAs/ZnSe QDs synthesized following our recently developed protocol^5^ based on commercially available tris-dimethylamino arsine (amino-As), alane *N,N*-dimethylethylamine as reducing agent, and ZnCl_2_ as additive. ZnCl_2_ plays a double role: (i) it improves the size distribution of InAs QDs, acting as a Z-type ligand, and (ii) it enables the in situ overgrowth of a thin ZnSe shell on the InAs QDs, thanks to the formation of an In-Zn-Se interlayer at the interface (see Figure S1 for QDs characterization).

Figure 1a presents a schematic of the fabricated LEDs and a scanning electron microscopy (SEM) image of the champion device (i.e., the device presenting the highest EQE). The champion device architecture comprises a thin layer (∼35 nm) of PEDOT:PSS deposited onto an indium tin oxide (ITO) pre-patterned substrate (Figure 1b). A 25 nm thick poly(N,N′-bis-4-butylphenyl-N,N′-bisphenyl)benzidine (poly-TPD) layer was spin-coated on the PEDOT:PSS, thus completing the hole injection and transport side of the architecture. The InAs/ZnSe QD film was deposited via spin-coating on top of poly-TPD, and the obtained layered structure was transferred into a thermal evaporator where TPBi, LiF, and Al layers were deposited. The LEDs were designed based on the ultraviolet photoelectron spectroscopy (UPS) analysis of InAs/ZnSe core/shell and InAs core-only QDs (Figures S2 and S3). The flat band energy diagram is reported in Figure 1c, and values for ITO, PEDOT:PSS, poly-TPB, TPBi, and LiF/Al were taken from the literature.^9^ The highest occupied molecular orbital of poly-TPD matches very closely the valence band maximum of the InAs/ZnSe QD film. Therefore, we do not expect a considerable energy barrier for the injection of holes into the active layer. On the other hand, the flat band diagram suggests a small energy barrier of 0.4 eV for electrons at the TPBi/QD film interface. Yet, such energy barrier is due to the thin ZnSe shell,^5^ whereas a favorable alignment between the lowest unoccupied molecular orbital of TPBi and the conduction band maximum of the InAs core is observed.

**Figure 1:**
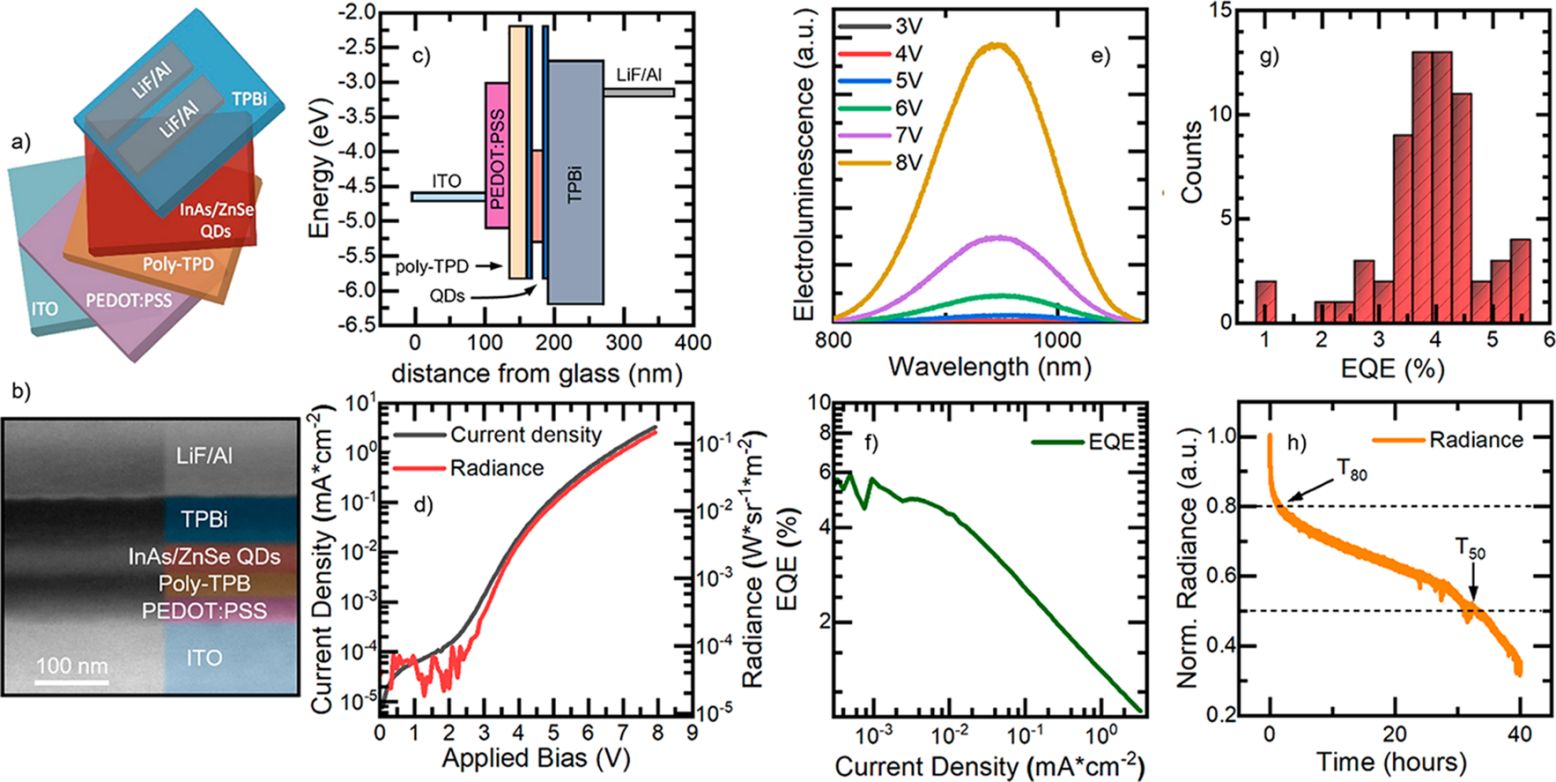
(a) Schematic, (b) SEM cross-sectional image, (c) flat energy-level diagram as a function of the device thickness, (d) current density (gray solid curve) and radiance (red solid curve) versus applied bias, (e) EL spectra at increasing applied bias, (f) EQE vs current density, (g) histogram of the EQEs of 64 pixels, and (h) stability test of the champion NIR LED.

The current density and radiance curves vs applied bias (JVR) for the champion device are reported in Figure 1d. The LED has a turn-on voltage of 2.4 V (estimated at a radiance of 8.17 × 10^–5^ W·sr^–1^·m^–2^) which is relatively high compared to the emission wavelength of the LED (Figure 1e, λ_EL_ = 947 nm, 1.31 eV). Noticeably, the LED features a limited leakage current (6.26 × 10^–5^ mA·cm^–2^ at 1 V), thus indicating the lack of parasitic channels which can have a detrimental impact on the device efficiency. The champion device has a maximum radiance of 0.15 W·sr^–1^·m^–2^ at 8 V; such radiance value is still low compared to the best PbS (9 W·sr^–1^·m^–2^)^10^ or In(Zn)As/In(Zn)P/GaP/ZnS (8.2 W·sr^–1^·m^–2^)^8^ QD LEDs. Yet, our QD LED shows a reduced current density with respect to those devices.^8,10^ Such reduced current could originate from the relatively thick layers employed in the champion LED demonstrating the highest EQE as reported for InP-based LEDs as well.^11^ On the other hand, poly-TPD has a hole mobility of 1 × 10^–4^ cm^2^·V^–1^·s^–1^,^9^ and we expect a reduced electron and hole mobility in the InAs/ZnSe QD layer considering the presence of long-chain ligands (oleylamine ∼2.5 nm).^12^ Overall, the JVR of our LEDs indicates that the fabricated devices are quite resistive (high turn-on voltage and low maximum current density), and improving further the conductivity could lead to higher radiance in the future. The EL spectra at increasing applied bias (Figure 1e) evidence a clear band-edge EL at 947 nm with a fwhm of 119 nm. As expected, the EL spectrum of our QD LEDs is red-shifted with respect to the photoluminescence (PL) (947 nm vs 931 nm, Figure S4). The champion device has a maximum EQE of 5.5% (Figure 1f). Importantly, while this manuscript was under review, an article by Zhao et al. appeared online demonstrating an EQE of 13.3% for InAs QDs synthesized via a tris(trimethylsilyl)arsine (TMS-As) route.^13^ The EQE from our champion device drops to ∼1% at the maximum current density. Yet, such radiance roll-off is similar to that of state-of-the-art NIR LEDs.^2,4^ The average maximum EQE calculated from 64 different pixels (Figure 1g) is 3.9%, only 30% lower than that of the champion device, thus underlining the reproducibility of the discussed results. The functional stability of LEDs is also an important figure of merit, and hybrid devices embedding organic layers and colloidal QDs often have a limited lifetime.^14^ Indeed, many detrimental phenomena can occur during driving of the LED.^15^ We tested the functional stability of a typical NIR LED by applying a constant current of 1 μA (corresponding to the maximum EQE, Figure 1h) for over 40 h in air without any LED encapsulation. The radiance shows a fast drop during the first hour of operation (20% drop, *T*_80_ = 1.34 h) after which the decrease is less sustained. In fact, it requires 32 h to reach 50% (*T*_50_) of the initial radiance value. This is an improved operational stability compared to the literature.^8^ We can tentatively attribute the durability of our LEDs to the rational device optimization and the high stability of the InAs/ZnSe QD layer.

The LEDs discussed in Figure 1 are based on the best-performing LED architecture we have identified. Poly-TPD was employed as the hole transport layer (HTL) as it leads to an improved EQE compared to other standard HTL materials. For example, when employing diphenylamine (TFB) as the HTL, the maximum EQE we could reach was 4.2%, with a TPBi thickness of 80 nm (Figure S5). In addition, we found that the thickness of the TPBi layer plays a crucial role in the final device performance for both TFB and poly-TPD HTLs (Figure S6).

In conclusion, we demonstrated efficient, stable, and fully RoHS-compliant NIR LEDs based on InAs/ZnSe QDs. Thanks to the rational device design and efficient QDs, we achieved an EQE of 5.5% and a corresponding radiance of 0.15 W·sr^–1^·m^–2^ at 947 nm. Our results demonstrate that InAs QDs prepared via amino-As route have reached a level of development that allows their exploitation in efficient NIR light sources. The devices presented here are only the first example of efficient NIR LEDs based on InAs QDs, and future development of more complex device architectures, as well as improvements in the QD synthesis will lead to more efficient RoHS-compliant NIR LEDs.

## Supporting Information Available

Experimental methods, InAs/ZnSe QDs characterization, and additional data on LEDs performance, including Figures S1–S6 (PDF)

## References

[ref1] Vasilopoulou M.; Fakharuddin A.; Garcia de Arquer F. P.; Georgiadou D. G.; Sargent E. H.; et al. Advances in solution-processed near-infrared light-emitting diodes. Nat. Photonics 2021, 15, 656 10.1038/s41566-021-00855-2.

[ref2] Gao L.; Quan L. N.; García de Arquer F. P.; Zhao Y.; Sargent E. H.; et al. Efficient near-infrared light-emitting diodes based on quantum dots in layered perovskite. Nat. Photonics 2020, 14, 227 10.1038/s41566-019-0577-1.

[ref3] Gong X.; Yang Z.; Walters G.; Comin R.; Sargent E. H.; et al. Highly efficient quantum dot near-infrared light-emitting diodes. Nat. Photonics 2016, 10, 253 10.1038/nphoton.2016.11.

[ref4] Vasilopoulou M.; Kim H. P.; Kim B. S.; Papadakis M.; bin Mohd Yusoff A. R.; et al. Efficient colloidal quantum dot light-emitting diodes operating in the second near-infrared biological window. Nat. Photonics 2020, 14, 50 10.1038/s41566-019-0526-z.

[ref5] Zhu D.; Bellato F.; Bahmani Jalali H.; Di Stasio F.; Prato M.; et al. ZnCl_2_ Mediated Synthesis of InAs Nanocrystals with Aminoarsine. J. Am. Chem. Soc. 2022, 144, 10515 10.1021/jacs.2c02994. 35648676 PMC9204758

[ref6] Jiao M.; Portniagin A. S.; Luo X.; Jing L.; Han B.; Rogach A. L. Semiconductor Nanocrystals Emitting in the Second Near-Infrared Window: Optical Properties and Application in Biomedical Imaging. Adv. Opt. Mater. 2022, 10, 2200226 10.1002/adom.202200226.

[ref7] Lu H.; Carroll G. M.; Neale N. R.; Beard M. C. Infrared Quantum Dots: Progress, Challenges, and Opportunities. ACS Nano 2019, 13, 939 10.1021/acsnano.8b09815. 30648854

[ref8] Wijaya H.; Darwan D.; Zhao X.; Ong E. W. Y.; Tan Z. K.; et al. Efficient Near-Infrared Light-Emitting Diodes based on In(Zn)As-In(Zn)P-GaP-ZnS Quantum Dots. Adv. Funct. Mater. 2020, 30, 1906483 10.1002/adfm.201906483.

[ref9] De Franco M.; Cirignano M.; Cavattoni T.; Jalali H. B.; Prato M.; Di Stasio F. Facile purification protocol of CsPbBr_3_ nanocrystals for light-emitting diodes with improved performance. Opt. Mater.: X 2022, 13, 100124 10.1016/j.omx.2021.100124.

[ref10] Pradhan S.; Di Stasio F.; Bi Y.; Gupta S.; Konstantatos G.; et al. High-efficiency colloidal quantum dot infrared light-emitting diodes *via* engineering at the supra-nanocrystalline level. Nat. Nanotechnol. 2019, 14, 72 10.1038/s41565-018-0312-y. 30510279

[ref11] Jalali H. B.; Sadeghi S.; Dogru Yuksel I. B.; Onal A.; Nizamoglu S. Past, present and future of indium phosphide quantum dots. Nano Res. 2022, 15, 4468 10.1007/s12274-021-4038-z.

[ref12] Ghosh S.; Das K.; Chakrabarti K.; De S. Effect of oleic acid ligand on photophysical, photoconductive and magnetic properties of monodisperse SnO_2_ quantum dots. Dalton Trans. 2013, 42, 3434 10.1039/C2DT31764H. 23258710

[ref13] Zhao X.; Lim L. J.; Ang S. S.; Tan Z. K. Efficient Short-Wave Infrared Light-Emitting Diodes based on Heavy-Metal-Free Quantum Dots. Adv. Mater. 2022, 2206409 10.1002/adma.202206409. 36097727

[ref14] Guo B.; Lai R.; Jiang S.; Zhou L.; Wang Y.; et al. Ultrastable near-infrared perovskite light-emitting diodes. Nat. Photonics 2022, 16, 637 10.1038/s41566-022-01046-3.

[ref15] Moon H.; Lee C.; Lee W.; Kim J.; Chae H. Stability of quantum dots, quantum dot films, and quantum dot light-emitting diodes for display applications. Adv. Mater. 2019, 31, 1804294 10.1002/adma.201804294. 30650209

