# Supplementary material for: Near-Infrared Light-Emitting Diodes Based on RoHS-Compliant InAs/ZnSe Colloidal Quantum Dots
Source: ACS Energy Lett. 2022 Oct 6;7(11):3788–90. doi: 10.1021/acsenergylett.2c02070 (PMC9664446; doi:10.1021/acsenergylett.2c02070)
Supplement: Supplementary file 1 — nz2c02070_si_001.pdf [file nz2c02070_si_001.pdf]

SUPPORTING INFORMATION

Near-Infrared Light-Emitting Diodes Based on RoHS-compliant InAs/ZnSe Colloidal Quantum Dots

Manuela De Franco[1,2,#], Dongxu Zhu[3,#], Aswin Asaithambi[4], Mirko Prato[5], Eleftheria Charalampous[6,7], Sotirios Christodoulou[6], Ilka Kriegel[4], Luca De Trizio[3], Liberato Manna[3], Houman Bahmani Jalali[2,3*] and Francesco Di Stasio[2*]

[1]Dipartimento di Chimica e Chimica Industriale, Università degli Studi di Genova, Via Dodecaneso 31, 16146 Genova, Italy

[2]Photonic Nanomaterials, [3]Nanochemistry, [4]Functional Nanosystems, and [5]Materials Characterization Facility, Istituto Italiano di Tecnologia, Via Morego 30, 16163 Genova, Italy

[6]Inorganic Nanocrystals Laboratory, Department of Chemistry, University of Cyprus, 1678 Nicosia, Cyprus.

[7]Experimental Condensed Matter Physics Laboratory, Department of Physics, University of Cyprus, 1678 Nicosia, Cyprus

Methods

LED device fabrication: Indium tin oxide (ITO) pre-patterned substrates were cleaned and sonicated in water and detergent (Hellmanex III), then rinsed with water, acetone, and isopropanol for 10 minutes. The ITO surface was activated by oxygen plasma treatment at 100 W for 180 seconds. The PEDOT:PSS (AI4083, Ossila) has been filtered through a 0.45 µm filter and then spin-coated at 4000 rpm for 45 seconds onto the substrate and annealed in air at 140 °C for 20 minutes. The devices were transferred into a glove box where the poly-TPD (8 mg.mL^{-1} in chlorobenzene) was deposited at 2000 rpm for 45 seconds and sequentially annealed at 120 °C for 20 minutes. The InAs/ZnSe QDs solution (∼15 mg.mL^{-1}) was filtered with a 0.20 µm PTFE filter and spin-coated at 1500 rpm for 45 seconds. Finally, the layers of TPBi, LiF and the Al cathode were evaporated through thermal evaporator in ultra-high vacuum. The cathode area overlaps the ITO anode determining an active area of 4.5 mm^2.

EL measurements and LED performance analyses: The devices having area of 4.5 mm^2 were characterized by their current density and radiance as a function of the applied bias. All the devices were measured in air without any further encapsulation. A Keithely 2636 source-measure unit connected to the LED was used to apply the bias and measure the current flowing through the device. The emitted light was measured with a calibrated Gentec germanium-based photodetector (PH20-Ge-D0). The radiance (R) was calculated as following: $R = \frac{L_0}{s_2}(W.sr^{-1}.m^{-2})$ where s_2 is the emissive area, and L_0 is the radiant intensity. The radiant intensity is defined as the light power emitted from a source

in the solid angle unit. Since the source shows Lambertian profile, the following equation was used: $L_0 = P * \Omega = P * l^2/s_1 (W * sr^{-1})$ where, P is the emitted power in the forward direction perpendicular to the LED surface, and $\Omega = l^2/s_1$ is the solid angle between the emitting area and the photodetector (l^2 is the distance between the two, and s_1 is the photodetector area). The external quantum efficiency (EQE) is the number of emitted photons (N_p) in the forward direction in free space over the number of injected charge carriers (N_e): $EQE = \frac{N_p}{N_e}$. N_p is calculated as $N_p = \frac{\pi * P * l^2 * \lambda}{s_1 * hc}$, where λ is the emission wavelength, hc is the Plank's constant (h= 6.62×10^{-34}Js) and the speed of the light in vacuum (c= 3×10^8 m.s^{-1}) respectively. The injected charge carrier $N_e = \frac{I}{q}$, where I is the current flowing into the device, and q is the elementary charge constant (1.6×10^{-19} C). The stability test was carried out in air at a fixed applied current of 1 µA and the radiant power was measured using the Ge photodetector for 40 hours. The EL spectrum characterization was carried out with a customized optical setup. The EL light was directly coupled to a Czerny-Turner HRS-500 spectrometer (Princeton Instruments) to resolve the light spectrally. The spectrally resolved light was detected and read using PIXIS CCD camera and Lightfield software (Princeton Instruments).

Ultraviolet photoelectron spectroscopy (UPS): The UPS measurements were carried out with a Kratos Axis UltraDLD spectrometer, using a He I (21.22 eV) discharge lamp, on an area of 55 µm in diameter, at a pass energy of 10 eV and with a dwell time of 100 ms. A -9.0 V bias was applied to the sample to precisely determine the low-kinetic-energy (i.e., high-binding-energy) cutoff, as discussed by M. G. Helander et al.[1] The position of the valence band maximum *versus* the vacuum level was estimated by measuring the energy difference between the high- and the low-binding energy cutoffs, as reported in ref[2].

Synthesis of InAs/ZnSe core/shell QDs: Indium(III) chloride ($InCl_3$, 99.999%), zinc(II) chloride ($ZnCl_2$, 99.999%), alane N,Ndimethylethylamine complex solution (DMEA−AlH_3, 0.5 M solution in toluene), triethyloxonium tetrafluoroborate (Et_3OBF_4, 97%), oleylamine (OLAM, 98%), toluene (anhydrous, 99.8%), octane (anhydrous, ≥99%)ethanol (anhydrous, 99.8%) and hexane (anhydrous, 95%) were purchased from Sigma-Aldrich. Tris(dimethylamino)arsine (amino-As, 99%), selenium powder (Se, 99.99%), tri-n-octylphosphine (TOP, 97%) were purchased from Strem. All the chemicals were used without further purification. The arsenic precursor was prepared by dissolution of 0.2 mmol of amino-As in 0.5 mL of degassed OLAM at 40 °C for 5 min in a nitrogen filled glovebox. For 1 M TOP-Se precursor, 10 mmol of selenium powder was mixed with 10 mL of TOP in a nitrogen filled glovebox at 150 °C till solved then cool down to room temperature. For the synthesis of InAs/ZnSe QDs, 0.2 mmol of $InCl_3$, 4 mmol of $ZnCl_2$, and 5 mL of OLAM were degassed at 120 °C under vacuum for 1 h and then heated to 240 °C under nitrogen atmosphere. Then, the arsenic precursor was injected, followed by the injection of 1.2 mL of the DMEA-AlH_3 toluene solution. Then, the temperature was

increased to 300°C, after 15 min, the flask was cooled down to 90 °C. 1 mL of 1 M TOP-Se precursor was injected into the flask, and flask was heated up to 310 °C for 10 min. Then, the mixture was transferred into glovebox, and added toluene for dispersion.

QDs purification: The QDs were purified by using ethanol as the anti-solvent in a precipitation-redispersion procedure for three times. Typically, 1mL of QDs solution were mixed with around 1mL of ethanol, as the QDs become less stable and begin to precipitate, the dispersion changes from a dark brown, limpid tint to a light brownish, turbid solution. The dispersion was centrifuged at 3000 rpm for six minutes, the supernatant was discarded, and the QDs pellet was thoroughly mixed with 1mL of toluene. At the last cycle, the precipitate was dissolved in 0.5 mL of octane for LED fabrication, or in toluene for other characterizations.

TEM and SEM measurements: Bright-field TEM images of the QDs were acquired by a JEOL JEM-1400 Plus transmission electron microscope equipped with thermionic source (LaB6) operating at an acceleration voltage of 120 kV. The images were acquired by Gatan CCD camera Orius 830 (2048 × 2048 active pixels). Sample was prepared by drop-casting onto carbon film-coated 200 mesh copper grids a diluted QD dispersion. The cross-section was performed via a FEI Helios NanoLab DualBeam 650 system (a scanning electron microscope/ focused ion beam workstation) with the Schottky FESEM column (Elstar™) and the 30 kV Ga Focused Ion Beam (Tomahawk™) placed at 52°one with respect to the other.

Optical absorption and PL measurements: The visible and NIR optical absorption spectrum of a diluted QD dispersion in toluene was collected with an Agilent Cary 5000 spectrophotometer. Steady-state PL measurements were performed with Acton750i Princeton spectrometer coupled with PIXIS (CCD) camera. All the samples were excited with 405 nm diode laser (power density = 0.03 $W.cm^{-2}$). The PLQY measurements were carried out with a 4-inch integrating sphere (Labsphere QE-060-SF) excited at 405 nm (power density = 0.13 $W.cm^{-2}$) and corrected for the system response function while we employed the method developed by J.C. de Mello et al [3].

X-ray Diffraction: The XRD analysis was carried out on a PANalytical Empyrean X-ray diffractometer, equipped with a 1.8 kW CuKα ceramic X-ray detector operating at 45 kV and 40 mA. Samples for the measurements were prepared by drop-casting a concentrated solution of QDs on a zero- diffraction silicon substrate.

InAs/ZnSe colloidal QDs characterization

The InAs/ZnSe QD dispersion has an excitonic absorption peak at ~857 nm, a PL peak at ~931 nm with full width at half maximum (fwhm) of 139 nm (210 meV) and a PLQY of 43±5% (Fig. S1a). The QDs have cubic zinc-blende crystal structure (Fig. S1b) with narrow particle size distribution (5.9±0.5 nm, Fig. S1c).

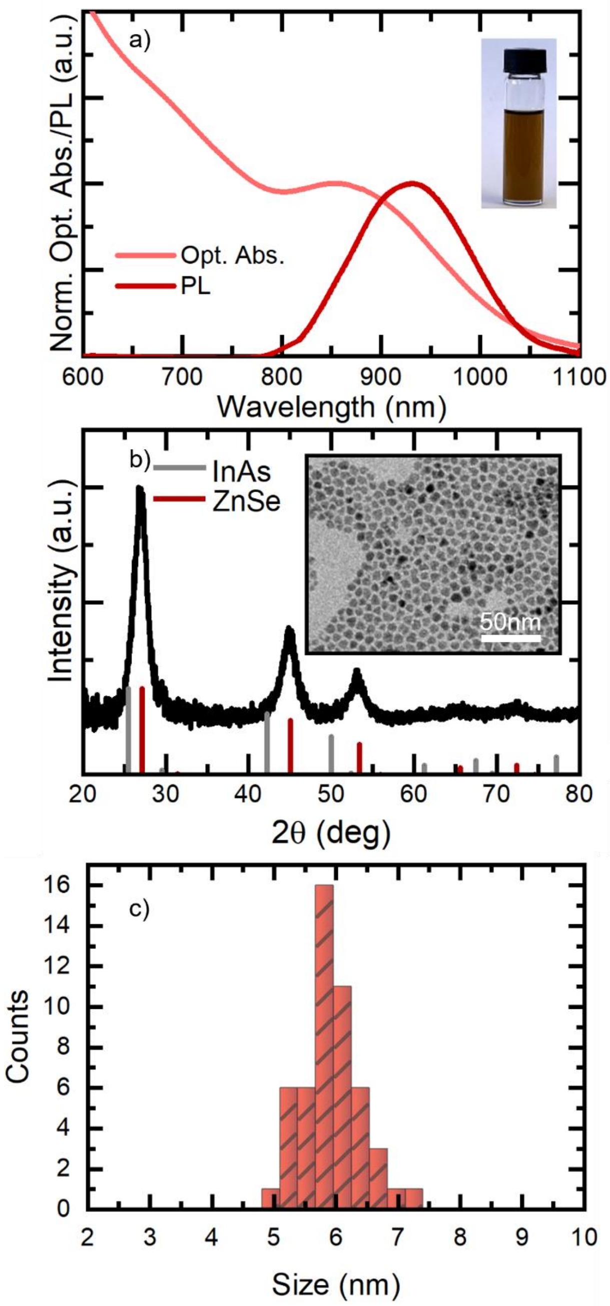

Figure S1 Optical and structural characterization of colloidal InAs/ZnSe QDs: (a) steady-state optical absorption (solid light red curve) and PL (solid dark red curve) of the InAs/ZnSe QD dispersion (inset: image of a QD dispersion under ambient light). (b) XRD patterns with the corresponding reflection of bulk zinc blende InAs (ref. 98-002-4518, solid grey bars) and ZnSe (ref. 98-007-7092, solid red bars), inset: TEM images of the colloidal InAs/ZnSe QDs. (c) Particle diameter distribution of the synthesized InAs/ZnSe core-shell QDs.

Ultraviolet Photoelectron spectroscopy (UPS) of InAs/ZnSe quantum dots

Fig. S2 reports the UPS data collected on InAs core only (Fig. S2a) and InAs/ZnSe core-shell QDs (Fig. S2b) employed for the LED fabrication. As reported in reference[2], from the distance between the high- and the low- binding energy onsets (whose positions were determined by linear fitting, as indicated in Fig. S2 by blue and red segments), we could estimate the position of the valence band maximum (VBM) with respect to the vacuum level. The obtained values for InAs/ZnSe core-shell and InAs core only QDs are -5.8±0.2 eV and -5.3±0.2 eV, respectively. Using optical bandgap values of 1.3 eV for InAs (extracted from the absorption spectrum in Fig. S1) and 3.5 eV for ZnSe (estimated from references[4, 5] considering a ZnSe thickness of 1.5 nm), we could build a flat energy diagram of the QDs (Fig. S3).

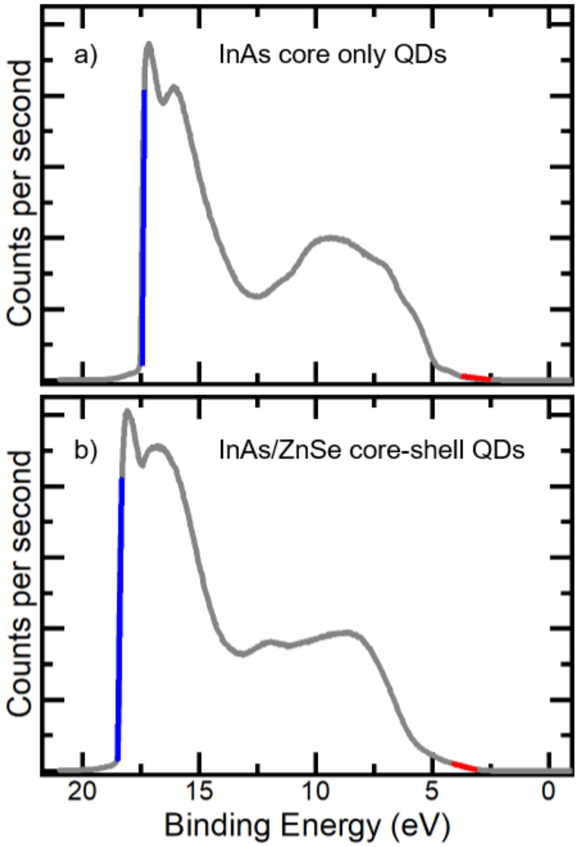

Figure S2 (a) Representative UPS spectrum of InAs core only QDs and (b) InAs/ZnSe core-shell QDs spin-cast films on a silicon wafer. For each sample, the energy difference between the two photoemission onsets (at high and low binding energy) has been determined and then subtracted from the energy of the used photons (21.22 eV), to obtain the position of the valence band maximum (VBM) with respect to the vacuum level.

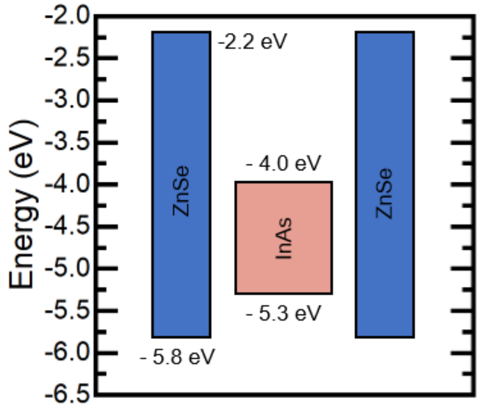

Figure S3 Flat energy-level diagram of InAs/ZnSe core-shell QDs spin-cast films on a silicon wafer. The valence band maxima and conduction band minima are obtained from the UPS spectra of figure S2 and from the estimated optical band gaps.

Electroluminescence and photoluminscence spectra comparison

In Fig. S4 we report the EL and the PL spectrum of the InAs/ZnSe QDs. The PL shows a maximum at 931 nm with a FWHM of 139 nm. On the other hand, the EL from our LEDs has a peak at 947 nm and a FWHM of 122 nm. Such EL red-shift is commonly observed in QD LEDs[6] and it is caused by the excitation process in electrically-driven emission as well as the high-density packing of the QDs in film. Furthermore, the EL spectra exhibited only a NIR peak arising from the QD layer, thus indicating that exciton formation takes place in the QD active layer.[7] Surprisingly, the EL presents a reduced FWHM compared to the PL (122 vs 139 nm, respectively); such difference can arise from the different spectrometers and the different conditions employed to record the two spectra (see methods) as well as from the increased self-absorption in a film of QDs.

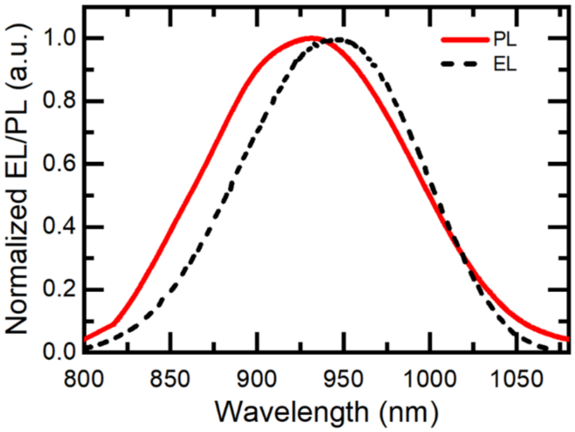

Figure S4 comparison between the EL from the InAs/ZnSe QD LEDs and the PL from the same QD dispersion.

Performance of LEDs based on TFB as HTL at increasing TPBi thickness

The best performing LED based on TFB as HTL had maximum radiance of 0.12 $W.sr^{-1}.m^{-2}$ at 8V and J_{max}= 2.4 $mA.cm^{-2}$ (Fig. S5). Nevertheless, considering the large library of hole or electron transport materials commercially available, it is important to underline that the EQE of 5.5% we present in the manuscript can be further improved in the future by testing other LED architectures.

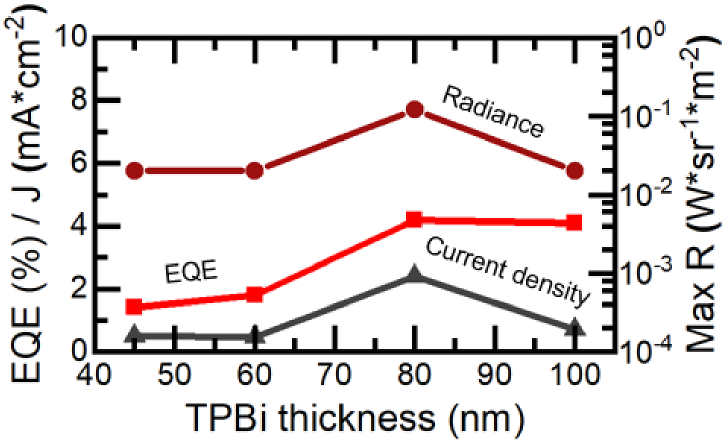

Figure S5 Variation of the EQE (red solid curve), current density (black solid curve) and maximum radiance (brown solid curve) vs the thickness of the TPBi ETL layer for LEDs with structure: ITO/PEDOT:PSS/TFB/QD film/TPBi/LiF/Al. The solid lines in the figure are guides for the eye.

Performance of LEDs based on poly-TPD as HTL at increasing TPBi thickness

As depicted in Fig. S6, increasing the TPBi layer thickness from 45 to 80 nm induces an increase in EQE as a side effect of the decrease in current density (while the radiance experiences only a minor improvement). By increasing the thickness of the ETL, one obtains the double effect of improving the electron supply to the active layer while enhancing the hole blocking capabilities of the latter. Yet, it is not trivial to assign the improved EQE to a single physical phenomenon as a variety of effects are at play. For example, the decrease in current density can be caused by an excess of holes in the active layer, which is mitigated by the improved electron supply, to a more efficient hole blockade (given the limited energy barrier of 0.7 eV the holes experience at the InAs/ZnSe QD - TPBi interface), or a shifting of the exciton recombination zone within the active layer. Increasing the TPBi thickness to 100 nm causes instead a drop in all device performance parameters.

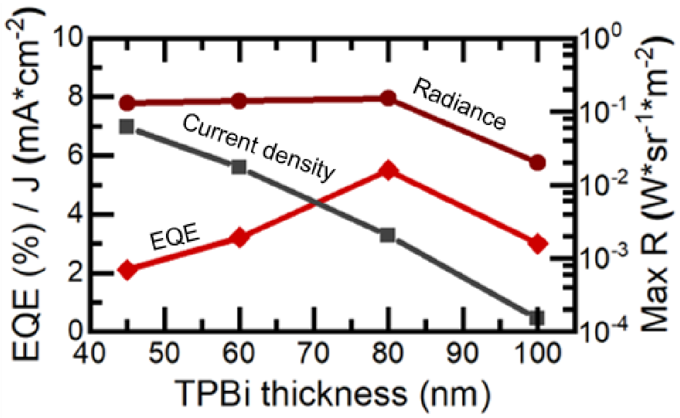

Figure S6 Variation of the EQE (red solid curve), current density (black solid curve) and maximum radiance (brown solid curve) vs the thickness of the TPBi ETL layer for LEDs with structure: ITO/PEDOT:PSS/poly-TPD/QD film/TPBi/LiF/Al. The solid lines in the figure are guides for the eye.

References

(1) Helander, M.; Greiner, M.; Wang, Z.; Lu, Z., Pitfalls in measuring work function using photoelectron spectroscopy. *Appl. Surf. Sci.* **2010,** *256* (8), 2602-2605.

(2) Tao, S.; Schmidt, I.; Brocks, G.; Jiang, J.; Tranca, I.; Meerholz, K.; Olthof, S., Absolute energy level positions in tin- and lead-based halide perovskites. *Nat. Commun.* **2019,** *10* (1), 1-10.

(3) de Mello, J. C.; Wittmann, H. F.; Friend, R. H., An improved experimental determination of external photoluminescence quantum efficiency. *Adv. Mater.* **1997,** *9* (3), 230-232.

(4) Cunningham, P. D.; Coropceanu, I.; Mulloy, K.; Cho, W.; Talapin, D. V., Quantized reaction pathways for solution synthesis of colloidal ZnSe nanostructures: a connection between clusters, nanowires, and two-dimensional nanoplatelets. *ACS Nano* **2020,** *14* (4), 3847-3857.

(5) Zhu, D.; Bellato, F.; Bahmani Jalali, H.; Di Stasio, F.; Prato, M.; Ivanov, Y. P.; Divitini, G.; Infante, I.; De Trizio, L.; Manna, L., $ZnCl_2$ Mediated Synthesis of InAs Nanocrystals with Aminoarsine. *J. Am. Chem. Soc.* **2022,** *144* (23), 10515-10523.

(6) Pradhan, S.; Di Stasio, F.; Bi, Y.; Gupta, S.; Christodoulou, S.; Stavrinadis, A.; Konstantatos, G., High-efficiency colloidal quantum dot infrared light-emitting diodes *via* engineering at the supra-nanocrystalline level. *Nat. Nanotechnol.* **2019,** *14* (1), 72-79.

(7) Anikeeva, P.; Madigan, C.; Halpert, J. E.; Bawendi, M.; Bulović, V., Electronic and excitonic processes in light-emitting devices based on organic materials and colloidal quantum dots. *Phys. Rev. B* **2008,** *78* (8), 085434.
